# Supplementary material for: Population Pharmacodynamic Models of Risperidone on PANSS Total Scores and Prolactin Levels in Schizophrenia
Source: Pharmaceuticals (Basel). 2024 Jan 23;17(2):148. doi: 10.3390/ph17020148 (PMC10891722; doi:10.3390/ph17020148)
Supplement: Supplementary file 1 [file pharmaceuticals-17-00148-s001.zip › pharmaceuticals-2808635-supplementary.pdf]

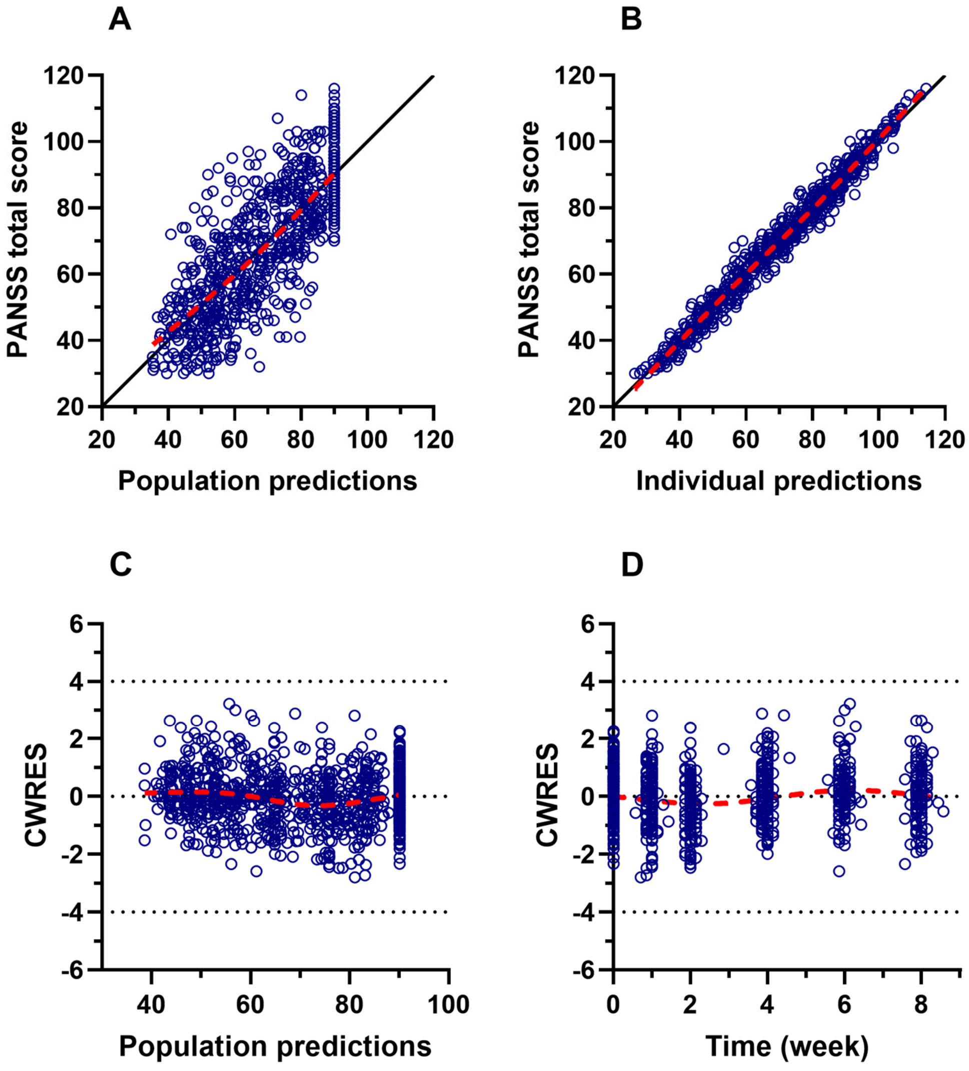

Supplemental Figure S1 Diagnostic plot of final population pharmacodynamic model of PANSS total scores. (A) Scatter plot of observations *vs.* population predictions. (B) Scatter plot of observations *vs.* individual predictions. (C) Scatter plot of conditional weighted residuals *vs.* population predictions. (D) Scatter plot of conditional weighted residuals *vs.* time after treatment. The black solid line is reference line. Red dotted line represents the LOESS line. CWRES conditional weighted residuals, PANSS positive and negative syndrome scale

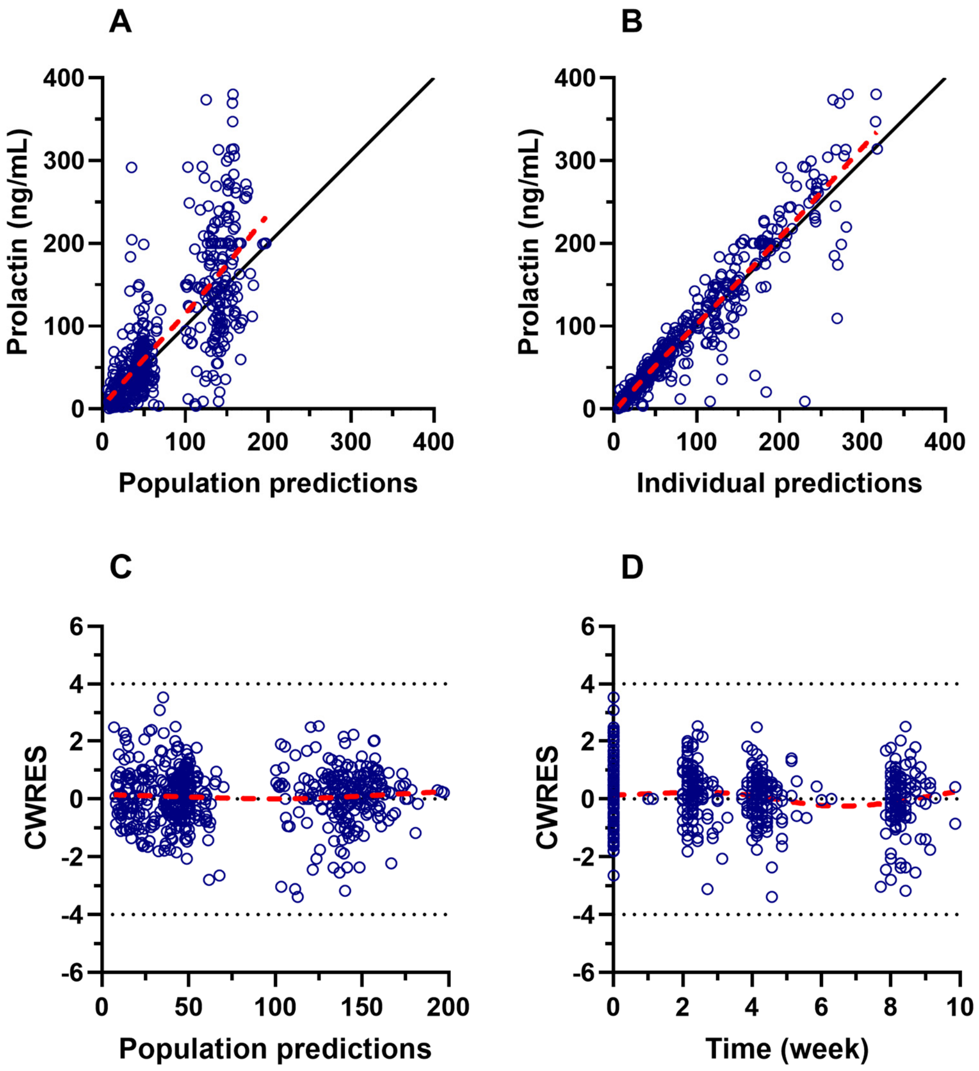

Supplemental Figure S2 Diagnostic plot of final population pharmacodynamic model of prolactin levels. (A) Scatter plot of observations *vs.* population predictions. (B) Scatter plot of observations *vs.* individual predictions. (C) Scatter plot of conditional weighted residuals *vs.* population predictions. (D) Scatter plot of conditional weighted residuals *vs.* time after treatment. The black solid line is reference line. Red dotted line represents the LOESS line. CWRES conditional weighted residuals

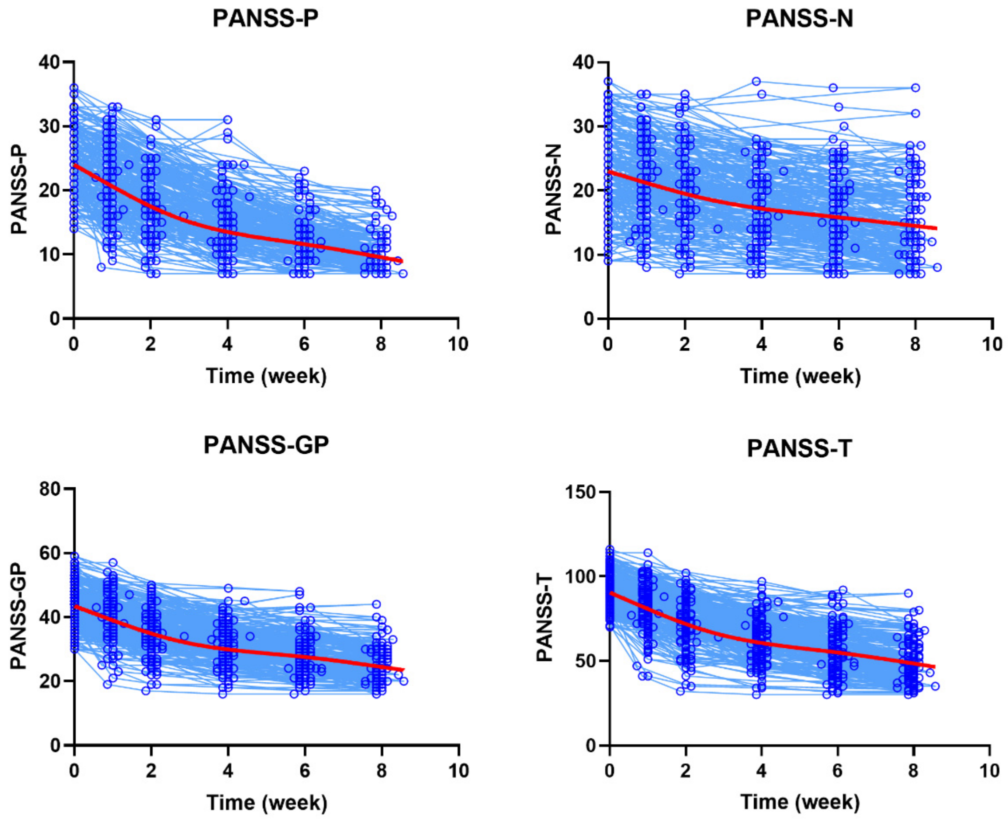

Supplemental Figure S3 Scatter plots of PANSS positive scores, PANSS negative scores, PANSS general psychopathology scores and PANSS total scores.

Red solid line represents the LOESS line. PANSS-P PANSS positive scores; PANSS-N PANSS negative scores; PANSS-GP PANSS general psychopathology scores; PANSS-T PANSS total score

List of clinical trial sites

1. Shanghai Mental Health Center, Shanghai, China
2. Beijing Huilongguan Hospital, Beijing, China
3. Guangzhou Huiai Hospital, Guangzhou, China
4. Hebei Provincial Mental Health Center, Baoding, China
5. Psychiatric Hospital of Henan Provincial, Xinxiang, China
6. Hunan Provincial Brain Hospital, Changsha, China
7. West China Hospital of Sichuan University, Chengdu, China
8. The First Hospital of Shanxi Medical University, Taiyuan, China
9. Tianjin Anding Hospital, Tianjin, China
10. Wuhan Mental Health Center, Wuhan, China
11. The First Affiliated Hospital of the Fourth Military Medical University of the People's Liberation Army of China, Chongqing, China
12. Shandong Provincial Mental Health Center, Jinan, China
